# Evaluation of the Conditions for the Cultivation of Callus Cultures of *Hyssopus officinalis* Regarding the Yield of Polyphenolic Compounds

**DOI:** 10.3390/plants10050915

**Published:** 2021-05-02

**Authors:** Olga Babich, Stanislav Sukhikh, Artem Pungin, Lidiya Astahova, Evgeny Chupakhin, Daria Belova, Alexander Prosekov, Svetlana Ivanova

**Affiliations:** 1 Institute of Living Systems, Immanuel Kant Baltic Federal University, A. Nevskogo Street 14, 236016 Kaliningrad, Russia; olich.43@rambler.ru (O.B.); stas-asp@mail.ru (S.S.); temon-aurum@mail.ru (A.P.); astahovalidiya@mail.ru (L.A.); chupakhinevgen@gmail.com (E.C.); antonina-daria@mail.ru (D.B.); 2 Laboratory of Biocatalysis, Kemerovo State University, Krasnaya Street 6, 650043 Kemerovo, Russia; a.prosekov@inbox.ru; 3 Natural Nutraceutical Biotesting Laboratory, Kemerovo State University, Krasnaya Street 6, 650043 Kemerovo, Russia; 4 Department of General Mathematics and Informatics, Kemerovo State University, Krasnaya Street 6, 650043 Kemerovo, Russia

**Keywords:** callus cultures, medicinal hyssop (*Hyssopus officinalis* L.), medicinal plants, biologically active substances

## Abstract

The cultivation of plants in the form of callus cultures constitutes a renewable source of secondary plant metabolites. The conditions for the cultivation of callus cultures affect the yield of target compounds. Callus cultures of *Hyssopus officinalis* were chosen for study. Nutrient media of various compositions were used for *Hyssopus officinalis* callus culture. For each culture, data on the quantitative contents of saponins, flavonoids and polyphenolic compounds, as well as antioxidant activity, were obtained. It was found that Murashige and Skoog medium supplemented with 1-naphthylacetic acid and kinetin led to the highest yield of secondary metabolites.

## 1. Introduction

The cultivation of plant tissue in the form of callus cultures is well understood [1,2]. Callus plant cultures constitute a renewable source of biologically active substances, which is independent of seasonal factors [3,4,5]. Obtaining biologically active substances from callus cultures is an area of interest because refining the nutrient medium can stimulate the biosynthesis of target biologically active substances and increase their yield [6,7]. The production of natural compounds via callus cultures of medicinal plants is promising in the development of industrial biotechnology for obtaining medically valuable substances [8]. The antioxidant activities and contents of polyphenolic compounds in medicinal plants in Siberia have been well studied, in a quantitative fashion [9,10,11]. An example of a well-studied plant is *H. officinalis* [12,13,14]. *Hyssopus officinalis* callus cultures were used to obtain styrenes and triterpenes [15]. In accordance with previous results, laboratory samples of *H. officinalis* callus were obtained [16], the chemical composition of *H. officinalis callus* used for the biotechnological production of phenolic compounds was studied, and the optimal composition of nutrient media was determined. In [17], the sequence of histogenesis and organogenesis in the in vitro callus of nodal explants of *H. officinalis* was studied upon treatment with growth regulators. This process is an important indicator of the ex vitro adaptability of regenerated seedlings. The rate of cell proliferation was enhanced in Murashige and Skoog medium supplemented with indole-3-butyric acid (1 mg/L). The study proceeded from the beginning of cultivation to the development of seedlings. Leaf and stem anatomy was influenced by growth regulators. Combinations of auxin and cytokinin aided the development of calli from regenerated shoots, revealing different degrees of vitrification in the stems and leaves. The influence of the cultivation conditions for callus cultures of *H. officinalis* on the yield of phenolic compounds was studied.

This study assessed the effects of the growing conditions for *H. officinalis* callus cultures on the yield of *H. officinalis* callus culture biomass and the accumulation of polyphenolic compounds.

## 2. Results

### 2.1. Callus Growth Curve

**Figure 1:**
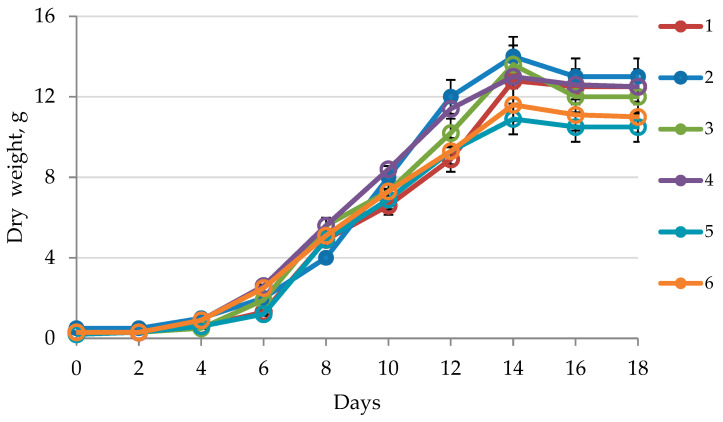
Growth curve of in vitro callus cultures of H. officinalis in various media: 1—MS-1 (medium MS + 500 mg/L Casein Hydrolyzate + 0.5 mg/L 6-benzylaminopurine (BA) + 2 mg/L 2,4-dichlorophenoxyacetic acid (2.4-D)); 2—MS-2 (medium MS + 2 mg/L Kin + 3 mg/L NAA); 3—MS-3 (medium MS + 1 mg/L BA + 2 mg/L indolylacetic acid (IAA)); 4—B5-1 (medium B5 + 500 mg/L Casein Hydrolyzate + 0.5 mg/L BA + 2 mg/L 2.4-D); 5—B5-2 (medium B5 + 2 mg/L Kin + 3 mg/L NAA); 6—B5-3 (medium B5 + 1 mg/L BA + 2 mg/L IAA). MS (Murashige-Skoog medium), B5 (Gamborg’s medium).

The growth curves of in vitro callus cultures in various media are presented in Figure 1. The greatest dry biomass weight of the H. officinalis callus cultures was observed on the 14th day of cultivation; by the 16th and 18th days, the amounts of dry biomass had slightly decreased. The greatest increase in the biomass of H. officinalis callus cultures was observed with MS-2 (medium Murashige–Skoog (MS) + 2 mg/L Kinetin (Kin) + 3 mg/L 1-naphthylacetic acid (NAA)) (up to 15 g of dry weight).

### 2.2. The Yield of Phenolic Compounds

For *H. officinalis*, which was cultivated on various nutrient media, the contents of polyphenolic compounds in the total extract were determined by high-performance liquid chromatography (HPLC) and are presented in Table 1. The highest yield of the phenolic compounds ferulic acid, isoquercitrin, rutin, quercetin, quercetin-7-O-glucoside and luteolin was observed in MS-2 (medium MS + 2 mg/L Kin + 3 mg/L NAA), at 31.15, 27.62, 19.75, 1.14, 0.67 and 1.98 µg/g, respectively (Table 1).

**Figure 2:**
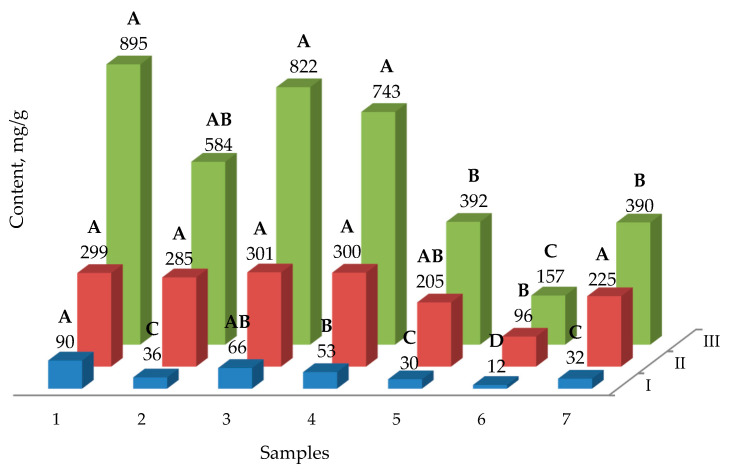
Phenolic compound (I—flavones; II—phenolic compound; III—2,2-diphenyl-1-pyridohydrazine (DPPH) antioxidant activity) contents of callus cultures of *Hyssopus officinalis* in different media cultivation: 1—control (native plants); 2—MS-1 (medium MS + 500 mg/L Casein Hydrolyzate + 0.5 mg/L BA + 2 mg/L 2.4-D); 3—MS-2 (medium MS + 2 mg/L Kin + 3 mg/L NAA); 4—MS-3 (medium MS + 1 mg/L BA + 2 mg/L IAA); 5—B5-1 (medium B5 + 500 mg/L Casein Hydrolyzate + 0.5 mg/L BA + 2 mg/L 2.4-D); 6—B5-2 (medium B5 + 2 mg/L Kin + 3 mg/L NAA); 7—B5-3 (medium B5 + 1 mg/L BA + 2 mg/L IAA). MS (Murashige–Skoog medium), B5 (Gamborg’s medium). Average values are presented (*n* = 3). Values followed by the same letter do not differ significantly (*p* > 0.05) as assessed by post hoc test (Tukey test).

For all the extracts of *H. officinalis,* the antioxidant activity, total phenolic compound content and total flavone content were determined (Figure 2).

### 2.3. The Yield of Saponins

**Figure 3:**
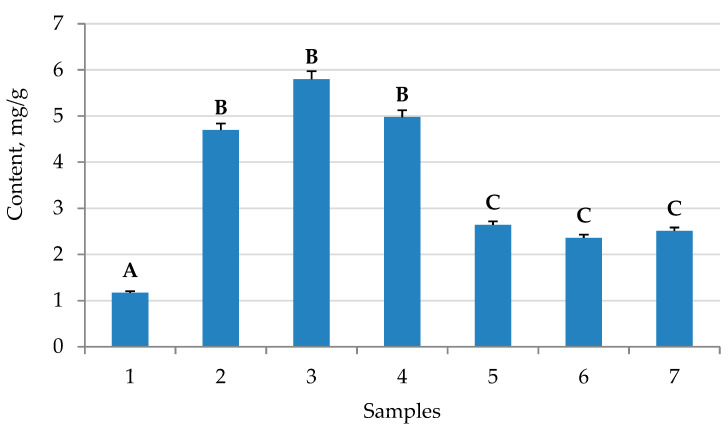
Saponins contents in callus culture of *Hyssopus officinalis* cultured in different media: 1—control (native plants); 2—MS-1 (medium MS + 500 mg/L Casein Hydrolyzate + 0.5 mg/L BA + 2 mg/L 2.4-D); 3—MS-2 (medium MS + 2 mg/L Kin + 3 mg/L NAA); 4—MS-3 (medium MS + 1 mg/L BA + 2 mg/L IAA); 5—B5-1 (medium B5 + 500 mg/L Casein Hydrolyzate + 0.5 mg/L BA + 2 mg/L 2.4-D); 6—B5-2 (medium B5 + 2 mg/L Kin + 3 mg/L NAA); 7—B5-3 (medium B5 + 1 mg/L BA + 2 mg/L IAA). MS (Murashige–Skoog medium), B5 (Gamborg’s medium). Values followed by the same letter do not differ significantly (*p* > 0.05) as assessed by post hoc test (Tukey test).

The influence of cultivation conditions on the yield of saponins is shown in Figure 3. The total saponins content was determined by the TLC method. The greatest quantity of saponins was found in *H. officinalis* callus cultures cultivated in MS-2 (medium MS + 2 mg/L Kin + 3 mg/L NAA) medium (5.8 mg/g).

## 3. Discussion

Our statistical analysis (Figure 1) showed no statistically significant differences in the growth rates of callus cultures based in MS media. Significant differences (*p* < 0.05) were observed in the samples cultivated in B5 media. The in vitro growth rates of callus cultures in B5-1 (medium B5 + 500 mg/L Casein Hydrolyzate + 0.5 mg/L BA + 2 mg/L 2.4-D) differed from the growth rates in B5-2 (medium B5 + 2 mg/L Kin + 3 mg/L NAA) and B5-3 (medium B5 + 1 mg/L BA + 2 mg/L IAA), and were statistically comparable to the samples cultivated in MS media throughout the observation period. No statistically significant differences arose for samples cultivated in MS-1 (medium MS + 500 mg/L Casein Hydrolyzate + 0.5 mg/L BA + 2 mg/L 2.4-D) or B5-1 (medium B5 + 500 mg/L Casein Hydrolyzate + 0.5 mg/L BA + 2 mg/L 2.4-D). It is possible that the absence of casein hydrolyzate in the B5-2 (medium B5 + 2 mg/L Kin + 3 mg/L NAA) and B5-3 (medium B5 + 1 mg/L BA + 2 mg/L IAA) cultivation media prevented similar parameters from being realized in these callus culture samples.

Many elements of nutrient media used for growing plant cultures determine the plants’ rates of growth and their accumulation of secondary metabolites. One of these metabolites is saponin, which accumulates in callus cultures. In all cases, an increased content of saponins in callus cultures compared to native plants was observed (Figure 3). In the case of cultivation on the nutrient medium MS-2 (medium MS + 2 mg/L Kin + 3 mg/L NAA), the highest yield of saponins was observed. The best characteristics of cell growth were also observed in MS-2 (medium MS + 2 mg/L Kin + 3 mg/L NAA). The lowest rate of cell growth was observed in the B5 medium. These effects are explained by the presence of different amounts of nutrients in the media. It was found that *H. officinalis* callus contains up to 6 mg/g of saponins, while intact plants contain six times less saponins. Callus plant cell cultures are increasingly being used as a tool for the synthesis of secondary metabolites. In intact plants, secondary metabolic pathways can be changed by external factors, such as the levels of nutrients, stress factors, light, and growth regulators. In callus cultures, the antioxidant activity was lower than in native plants. The yield of phenolic compounds was comparable to that in local plants, and the highest yield was observed in the MS-2 (medium MS + 2 mg/L Kin + 3 mg/L NAA) and MS-3 (medium MS + 1 mg/L BA + 2 mg/L IAA) growth media. The highest yield of flavones was found when using MS-2 (medium MS + 2 mg/L Kin + 3 mg/L NAA) and MS-3 (medium MS + 1 mg/L BA + 2 mg/L IAA) growth media. In the case of MS-2 (medium MS + 2 mg/L Kin + 3 mg/L NAA), 1-naphthylacetic acid was used as a growth promoter [18], along with kinetin [19]. From the obtained observation results, it was found that the most suitable medium, leading to a significant accumulation of flavonoids and saponins, was MS-2 (medium MS + 2 mg/L Kin + 3 mg/L NAA), with the addition of 1-naphthylacetic acid and kinetin as growth stimulants. Our results for the antioxidant activity of medicinal hyssop are consistent with previously published data [12]. In particular, the antioxidant activity of the MS-2 (medium MS + 2 mg/L Kin + 3 mg/L NAA) as assessed by DPPH had a medium value of 822 μg/mL, while this value is 796 μg/mL in the published data. Our data and the literature both determined the phytochemical composition of hyssop [20], and the following compounds were found: ferulic acid, rutin, quercetin, luteolin. A comparison of the contents of these compounds in callus cultures and native plants shows that a similar level of secondary metabolites was produced by callus cultures. The use of a nutrient medium allows callus crops to achieve values comparable with native plants. Typical cultivation conditions for callus cultures are used when the goal is to induce and increase the yield of flavonoids [14]. The study in [21] is a key work on this topic, wherein the growth stimulants 2,4-dichlorophenoxyacetic acid and 1 mg/L benzyladenine were used, which have demonstrated high efficiency. In our original study, it was found that in hyssop callus cultures, 1-naphthylacetic acid can more efficiently produce aromatic secondary metabolites. The use of kinetin as a growth promoter was previously reported to be effective [22]. Additionally, in our study, kinetin had a positive effect on the growth and production of secondary metabolites. A recent publication reported the synergistic effect of kinetin and 1-naphthylacetic acid [23]. The results (Figure 1, Figure 2 and Figure 3) suggest that the mineral composition of the medium is the most important factor in the production of phenol and saponins. NAA–kinetin is thought to enhance the production of these compounds for MS media, but not B5. A significant influence of the medium’s mineral composition and the presence of casein hydrolyzate on the production of biologically active substances has been established. This hypothesis may explain the observed effects, including the increase in the growth and production of flavanoids. It should also be noted that plant cultivation and the isolation of individual flavonoids can be scaled up to the industrial scale. An example of this shift from the laboratory to the industrial scale is discussed in [24]. In our study, we achieved an efficient separation of flavanoids, which allows for their further isolation via preparative chromatography methods. Similar examples have previously been discussed in the literature [25]. The proposed method for the cultivation of callus cultures of hyssop does not have any disadvantages associated with seasonal factors. Stimulating the production of flavonoids allows the use of available reagents for the design of culture media, and also allows for scaling the volume of callus culture under strictly standardized conditions. This fact is important in the context of the biotechnology market [26].

## 4. Materials and Methods

Medicinal plants (*Hyssopus officinalis* L., *Lamiaceae* family) collected in 2018 and obtained from the collection of the Gorno-Altai Botanical Garden (Russia, Altai Republic, Shebalinsky district, Kamlak village, Chisty Lug tract), and their callus cultures, were the object of the research. Callus cultures were studied after 14 days of in vitro cultivation, when the greatest amount of biologically active substances had accumulated. Ferulic acid (certified reference material, CAS: 537-98-4), isoquercitin (certified reference material, CAS: 482-35-9), rutin (certified reference material CAS: 153-18-4), quercetin (certified reference material CAS: 117-39-5), quercitin-7-O-glucoside (certified reference material CAS: 491-50-9), luteolin (certified reference material CAS: 491-70-3) and oleanolic acid (≥97%, O5504) were purchased from Fluka/Sigma-Aldrich (Sigma-Aldrich Rus, Moscow, Russia). All the other chemicals (analytical grade and above) used in this study were obtained from the Research Institute of Biotechnology, Kemerovo State University (Kemerovo, Russia).

### 4.1. Obtaining Plant Callus Cell Cultures

Young tissues from seedlings (hypocotyls, cotyledons) were used as explants to obtain in vitro callus cultures, being suitable for this than mature ones [27]. For the induction of callusogenesis, the mineral media MS (Murashige and Skoog basal medium, M5519, Sigma-Aldrich) and B5 (Gamborg’s B5 basal salt, G5768, Sigma-Aldrich mixture) [28] supplemented with growth regulators (Table 2) were used. In total, 0.7% agar-agar was used as a gelling agent.

Aseptic plants and cell cultures were obtained via pre-washing the seeds with detergent, which were then immersed for 1 min in a 75% ethanol solution, transferred to a laminar box and sterilized for 15 min in a 20% sodium hypochlorite solution (5% active chlorine). They were then washed for 20 min in distilled sterile water three times. The sterile seeds were placed in vessels filled with Murashige–Skoog medium (MS) [2], containing 3% sucrose and 0.7% agar-agar without growth stimulants (Murashige and Skoog Basal Medium, M9274, Sigma-Aldrich). For germination, the seeds were placed in a thermostat at a temperature of 24 ± 1 °C until sprouts appeared, after which the sprouts were transferred to a light cabinet with a photoperiod of 16 8^−1^ h at a temperature of 24 ± 2 °C. Sterile 14-day-old *H. officinalis* seedlings were used to obtain callus cultures. The nutrient media were sterilized via autoclaving (20 min in the main mode; temperature, 121 °C; additional pressure, 0.7–0.8 atmospheres).

### 4.2. Cultivation of Callus Cell Cultures

The cultivation of callus cell cultures was performed under sterile conditions, in the dark at 24 ± 1 °C (incubator/thermostat BD 53, Binder, Germany) and with a relative humidity of 60–70%. The subcultivation cycle for callus cultures was 28–35 days. The callus was divided into 2–3 parts, depending on the growth, and was transplanted to a medium of identical composition. Callus cultures of *H. officinalis* were obtained using the following culture media based on MS (Murashige and Skoog [29]): MS-1 (medium MS + 500 mg/L Casein Hydrolyzate + 0.5 mg/L BA + 2 mg/L 2.4-D), MS-2 (medium MS + 2 mg/L Kin + 3 mg/L NAA), MS-3 (medium MS + 500 mg/L Casein Hydrolyzate + 0.5 mg/L BA + 2 mg/L 2.4-D). In addition, the following based on B5 (Gamborg’s [30]) were used: B5-1 (medium B5 + 500 mg/L Casein Hydrolyzate + 0.5 mg/L BA + 2 mg/L 2.4-D), B5-2 (medium B5 + 2 mg/L Kin + 3 mg/L NAA), B5-3 (medium B5 + 1 mg/L BA + 2 mg/L IAA). The compositions of the media are presented in Table 2.

### 4.3. Study of Growth Characteristics of Callus Cell Cultures (*In Vitro*)

To determine the initial weight (of the transplant), the cultures were weighed before transplantation into a culture vessel (Petri dish) with the medium. After placing the transplant onto the medium, it was weighed again, with the weight of the transplant determined as the difference between the second and the first weighing. The weights of the transplants were equalized to an accuracy of ± 10%. Biomass growth was determined using standard gravimetric methods [31].

### 4.4. Drying of Callus Cell Culture Biomass

The callus cell culture biomass was dried via lyophilization (vacuum 0.05 mbar; cooler temperature −80 °C) using a Triad lyophilic drying unit (Labconco, Kansas City, MO, USA) for 15 h (sample temperature during drying –20 °C) [32].

### 4.5. Preparation of Extract Samples

Samples of lyophilically dried biomass were extracted with a solution of 60% methanol in water (1:30 ratio of biomass:extractant) under constant stirring in a thermoshaker (20 °C, 1400 rpm) for 40 min three times. After each interval, the extracts were centrifuged (20 °C, 4500 rpm, 10 min). The supernatant liquid was taken for the qualitative analysis of phytocomponents via HPLC [33].

### 4.6. Quantitative Determination of the Contents of Individual Phenolic Compounds by HPLC

All chromatograms were obtained on an HPLC Shimadzu module LC-10AD and a DAD SPD-M20A. Chromatographic separation was performed on an RP-18 chromatographic column. The chromatographic column has a C18 standby phase with a phenyl-like end group, which is 25 cm long, 0.4 cm in diameter and 5 μm particle size, connected to the chromatographic column pre-block. Mobile phase: A—water containing 1% trifluoroacetic acid (99:1, *v:v*) and solvent; B—100% methanol, flow rate 1 mL/min, gradient 5% of B, increasing to 70% of B over 45 min. The injection volume was 1 μL of callus extract; a Shimadzu SIL-20AC autosampler was used for injection. The UV spectra of various compounds were recorded in the range of 240 to 400 nm. Detection was performed at 280 and 320 nm. Peak identification was confirmed by comparing the peak retention time with that of a pure standard, and quantification was performed by comparing the peak area of the sample chromatogram with the peak area of the standard. All measurements were carried out three times at 40 °C, and the results were expressed as the mean ± micrograms of phenolic compounds/1 g standard deviation of plant material [20].

### 4.7. Determination of the Total Polyphenol Compounds

The modified Folin–Ciocalteu method was used to determine the total contents of plant polyphenols in gallic acid using a UV 3600 spectrophotometer (Shimadzu, Japan) [34]. This method involves the oxidation reaction of plant polyphenols with the Folin–Ciocalteu reagent, and the subsequent photometric determination of the blue complex obtained at a wavelength of 765 nm. Gallic acid was used as an internal standard.

### 4.8. Determination of Total Flavonoids

Total flavonoids (in terms of rutin) in the plants were determined using the spectrophotometric method with a UV 3600 spectrophotometer (Shimadzu, Kyoto, Japan). The method [35] involves the spectrophotometric detection of flavanol complexes with aluminum chloride at a wavelength of 410-413 nm. Rutin was used as the internal standard.

### 4.9. Qualitative and Quantitative Determination of Saponins

The total saponin levels in the plants were determined using thin-layer chromatography in the benzene–acetone system (3:1). Detection was performed with 10% sulfuric acid. Oleanolic acid was used as the witness (Sigma-Aldrich Rus, Moscow, Russia). The amount of triterpene saponin was determined by UV spectroscopy using a UV 3600 spectrophotometer (Shimadzu, Japan) [36] after reaction with concentrated sulfuric acid. A typical maximum absorption was observed at 310 nm. The content of biologically active substances in the cell cultures was compared with that in the intact plants. The ages of these plants depended on which parts were used to isolate phenols, saponins, and flavonoids (hypocotyls, cotyledons, immature embryos, inflorescences). The ages of the plants were 1–2 months.

### 4.10. Antioxidant Activity

The ability of antioxidants to trap 2,2-diphenyl-1-pyridohydrazine (DPPH) free radicals was used to determine the antioxidant activity of the plant extracts via spectrophotometry on a Shimadzu UV 3600 spectrophotometer [37]. This method was performed as described in [38]. Different volumes (200 μL, 400 μL and 800 μL) of different callus extracts were mixed, then Tris buffer (100 mM, pH 7.4) and 1 mL of DPPH (1,1-diphenyl-2-pyridohydrazine) (500 μM) were added to obtain a final volume of up to 2 mL. After 30 min incubation at room temperature, the formation of a yellow complex was determined via spectrophotometry at 517 nm. Tocopherol and ascorbic acid were used as the positive control, and the reaction mixture without the extract was used as the negative control.

### 4.11. Statistical Analysis

Each experiment was repeated three times and the data are expressed as means ± standard deviation. Data processing was carried out via the standard methods of mathematical statistics. The correspondence of the samples used to the normal distribution was assessed via t-test (mathematical expectations) for independent samples, and by Fisher’s test (variance). Post hoc analysis (Tukey test) was undertaken to identify samples that were significantly different from each other. The equality of the variances of the extracted samples was checked using the Levene test. The data were subjected to analysis of variance (ANOVA) using Statistica 10.0 (StatSoft Inc., 2007, Tulsa, OK, USA). Differences between means were considered significant when the confidence interval was below 5% (*p* < 0.05).

## 5. Conclusions

The potential of callus cell cultures of *Hyssopus officinalis* to produce antioxidant substances has been studied in different cultivation media. Under certain cultivation conditions, the *Hyssopus officinalis* callus cultures produced significant amounts of phenolic compounds, and these were the most abundant of all the biologically active substances in the callus cultures. The use of suitable cultivation media has resulted in significant contents of saponins in the callus culture extracts of *Hyssopus officinalis*. An effective medium for the cultivation of callus in our study was MS supplemented with 2 mg/L kinetin and 3 mg/L 1-naphthyl acetic acid as growth stimulants. In the future, this will presumably allow us to determine the mechanisms of the accumulation of secondary metabolites in plants under stress, and offer opportunities for managing the synthesis of unique bioactive components in rare medicinal plants, including the ones growing in Siberia and the Far East. Through our studies, we determined the optimal parameters of the nutrient medium used for the biotechnological cultivation of *H. officinalis*, which will allow the future scaling-up of the technology for obtaining phenolic compounds from callus cultures.

## Figures and Tables

**Table 1:** Phenolic compound content (µg/g, average of three repetitions) determined by HPLC in plant material.

Name of Phenolic Compound	Rt (min) ± 0.5	SD	NP	Culture Media					
				MS-1	MS-2	MS-3	B5-1	B5-2	B5-3
Ferulic acid	17.60	1.00	36.92	28.62	31.15	29.17	22.37	11.34	20.43
Isoquercitrin	27.80	0.50	32.78	22.83	27.62	25.14	17.13	9.76	19.71
Rutin	28.50	1.00	21.93	17.43	19.75	18.19	12.45	8.34	13.43
Quercetin	32.00	0.10	1.79	0.97	1.14	1.03	0.54	0.41	0.50
Quercetin-7-O-glucoside	35.20	0.10	0.89	0.45	0.67	0.57	0.24	<0.2	0.40
Luteolin	48.20	0.10	2.25	1.14	1.98	1.60	0.76	0.31	0.82

Rt—retention time; SD—standard deviation of Rt; NP—native plants. MS-1 (medium MS + 500 mg/L Casein Hydrolyzate + 0.5 mg/L BA + 2 mg/L 2.4-D); MS-2 (medium MS + 2 mg/L Kin + 3 mg/L NAA); MS-3 (medium MS + 1 mg/L BA + 2 mg/L IAA); B5-1 (medium B5 + 500 mg/L Casein Hydrolyzate + 0.5 mg/L BA + 2 mg/L 2.4-D); B5-2 (medium B5 + 2 mg/L Kin + 3 mg/L NAA); B5-3 (medium B5 + 1 mg/L BA + 2 mg/L IAA). MS (Murashige–Skoog medium), B5 (Gamborg’s medium). Culture medium compositions are presented in Table 2.

**Table 2:** Composition of culture media.

Components	MS-1	MS-2	MS-3	B5-1	B5-2	B5-3
Sucrose, g	30	30	30	30	30	30
Casein Hydrolyzate, mg	500	-	-	500	-	-
Myo-inositol, mg	100	100	100	100	100	100
Thiamine, mg	0.1	0.1	0.1	10.0	10.0	10.0
Pyridoxine, mg	0.5	0.5	0.5	1.0	1.0	1.0
Nicotinic acid, mg	0.5	0.5	0.5	1.0	1.0	1.0
Kinetin, mg	-	2	-	-	2	-
6-benzylaminopurine, mg	0.5	-	1.0	0.5	-	1.0
Indolylacetic acid, mg	-	-	2	-	-	2
1-naphthylacetic acid, mg	-	3	-	-	3	-
2,4-dichlorophenoxyacetic acid, mg	2.0	-	-	2.0	-	-

MS (Murashige–Skoog medium), B5 (Gamborg’s medium).

## Data Availability

Not applicable.
